# Periodontal disease and subsequent risk of cardiovascular outcome and all-cause mortality: A meta-analysis of prospective studies

**DOI:** 10.1371/journal.pone.0290545

**Published:** 2023-09-08

**Authors:** Xiangyu Guo, Xue Li, Chunjuan Liao, Xingyu Feng, Tao He

**Affiliations:** State Key Laboratory of Oral Diseases & National Center for Stomatology & National Clinical Research Center for Oral DiseasesWest China Hospital of Stomatology, Sichuan University, Chengdu, Sichuan, China; The University of Mississippi Medical Center, UNITED STATES

## Abstract

Studies reported periodontal disease (PD) periodontal disease is associated with many systemic diseases, including cardiovascular outcomes and all-cause mortality. However, the precise mechanistic link for these relationship remained unclear. We therefore performed a meta-analysis of cohort studies to investigate the association of PD with the risk of cardiovascular outcomes and all-cause mortality. We systematically searched the databases of PubMed, EmBase, and the Cochrane library to identify eligible studies until April 2023. The investigated outcomes included major adverse cardiovascular events (MACEs), coronary heart disease (CHD), myocardial infarction (MI), stroke, cardiac death, and all-cause mortality. The summary relative risk (RR) with 95% confidence interval (CI) were calculated using the random-effects model. Thirty-nine cohort studies with 4,389,263 individuals were selected for final meta-analysis. We noted PD were associated with elevated risk of MACEs (RR: 1.24; 95%CI: 1.15–1.34; *P*<0.001), CHD (RR: 1.20; 95%CI: 1.12–1.29; *P*<0.001), MI (RR: 1.14; 95%CI: 1.06–1.22; *P* = 0.001), stroke (RR: 1.26; 95%CI: 1.15–1.37; *P*<0.001), cardiac death (RR: 1.42; 95%CI: 1.10–1.84; *P* = 0.007), and all-cause mortality (RR: 1.31; 95%CI: 1.07–1.61; *P* = 0.010). Sensitivity analyses indicated the pooled conclusions for cardiovascular outcomes and all-cause mortality are robustness. The associations of PD with the risk of ardiovascular outcomes and all-cause mortality could affected by region, study design, PD definition, follow-up duration, and study quality. This study found the risk of cardiovascular outcomes and all-cause mortality were elevated in PD patients, and the intervention for PD should be applied to prevent the risk of cardiovascular outcomes.

## Introduction

Cardiovascular disease (CVD) accounted for 17.8 million deaths worldwide in 2017, and these CVD deaths were caused by ischemic heart disease and stroke were 50% and 35%, respectively [1]. Moreover, ischemic heart disease and stroke were the top-ranked causes for disability-adjusted life-years in patients aged 50 years or older [2]. Managing patients at risk of CVD are important for prevent hospital admissions, and improve the quality of life. Currently, the independent risk factors for CVD included age, smoking, obesity, hypertension, diabetes mellitus, reduced high-density lipoprotein (HDL), and elevated low density lipoprotein (LDL) [3–5]. An additional risk factor should be identified for primary and secondary prevention and improve the prognosis of CVD.

Periodontal disease (PD) is a chronic inflammatory disease, which caused by dental plaque through attacks the immune defense line of periodontal tissue. The clinical manifestations of PD included gingiva bleeding, periodontal pocket, alveolar bone resorption, and tooth loss. PD is highly prevalent accounting for 20–50% of the global population, which could cause a loss of clinical attachment, and the progressive of PD could induce the loosening of teeth or even to the loss of teeth [6]. Study have demonstrated PD it not only a common chronic infectious and inflammatory oral disease, but also associated with the risk of several systemic disease, including rheumatoid arthritis, diabetes mellitus, chronic obstructive pulmonary disease, depression, and Alzheimer’s disease [7–11]. Nowadays, more and more attention has been paid to the association of PD with the risk of cardiovascular outcome and all-cause mortality. Several systematic review and meta-analyses were performed to assess the association between PD with the risk of cardiovascular outcomes [12–15], whereas the exploratory analyses for these relationship remained unclear. Therefore, we conducted this systematic review and meta-analysis to evaluate the association of PD with the risk of cardiovascular outcome and all-cause mortality.

## Materials and methods

### Literature search and selection criteria

The Meta-analysis Of Observational Studies in Epidemiology protocol was applied to perform and report this systematic review and meta-analysis [16]. Cohort study assess the association of PD with the risk of cardiovascular outcome and all-cause mortality was potential eligible in this study, and the publication language and status were not restricted. The databases of PubMed, EmBase, and the Cochrane library were systematically searched for eligible studies from their inception until April 2023, and using the following search terms: ("Periodontal disease" OR "tooth loss" OR "missing teeth" OR "periodontal") AND ("cardiovascular disease" OR "atrial fibrillation" OR "heart failure" OR "stroke" OR "cerebrovascular" OR "angina" OR "acute coronary syndrome"). We also manually reviewed the reference lists of relevant original article and review to identify any new study met the inclusion criteria.

The literature search and study selection were independently performed by 2 reviewers following the standardized flow, and conflicts between reviewers was resolved by discussed with a third reviewer until a consensus was reached. The details of inclusion criteria included: (1) Participants: adult population free of CVD at initially; (2) exposure: moderate or severe PD; (3) Control: individuals without PD or mild PD; (4) Outcomes: the study should reported at least 1 of major adverse cardiovascular events (MACE), coronary heart disease (CHD), myocardial infarction (MI), stroke, cardiac death, and all-cause mortality; and (5) Study design: cohort study, irrespective prospective or retrospective cohort design. There was no restriction was placed on the minimum number of participants. The study was excluded if they met: (1) cross-sectional or case-control design; (2) the effect estimate was presented without 95% confidence intervals (CIs); or (3) study did not reported cardiovascular outcome or all-cause mortality.

### Data extraction and quality assessment

Two reviewers independently performed data collection, and inconsistent results between reviewers was settled by a third reviewer referring to full-text of article. The abstracted information in each included study included first authors’ surname, publication year, region, study design, sample size, range of individuals’ age, male proportion, the definition of PD, reported outcomes, follow-up duration, and adjusted factors. The Newcastle-Ottawa Scale (NOS) was used to assess the methodological quality of observational studies in meta-analysis, which including selection (4 items), comparability (1 item), and outcome (3 items) domains, and the “star system” in individual study ranged from 0–9. Study with 7–9 stars was regarded as high quality, 4–6 stars was defined as moderate quality, and with 0–3 stars was considered as low quality [17]. The quality assessment was independently performed by 2 reviewers, and an additional reviewer was examined and adjudicated disagreement between reviewers through referring to the original article.

### Statistical analysis

The association of PD with the risk of cardiovascular outcomes and all-cause mortality based on the effect estimate with 95%CI in individual study. The summary relative risk (RR) and 95%CI were calculated using the random-effects model with Mantel-Haenszel Statistics, which considering the underlying varies across included studies [18,19]. The *I*^*2*^ and Cochren Q statistic was applied to assess the heterogeneity across included studies, and *I*^*2*^ ≥ 50.0% or *P* < 0.10 was defined as significant heterogeneity [20,21]. Sensitivity analysis was applied to assess the robustness of pooled conclusion through sequential removing single study [22]. Subgroup analyses were conducted based on region, study design, gender, PD definition, follow-up duration, adjusted level, and study quality, and the differences between subgroups were assessed using interaction *P* test, which assuming the data met the normal distribution [23]. Publication bias were assessed using both qualitative and quantitative methods, including funnel plot, Egger, and Begg tests [24,25]. The *P* value for pooled conclusions are 2-sided, and *P* < 0.05 was considered as statistically significant. Statistical analysis in our study was performed by using STATA software (version 12.0; Stata Corporation, College Station, TX, USA).

## Results

### Search of the literature

**Fig 1:**
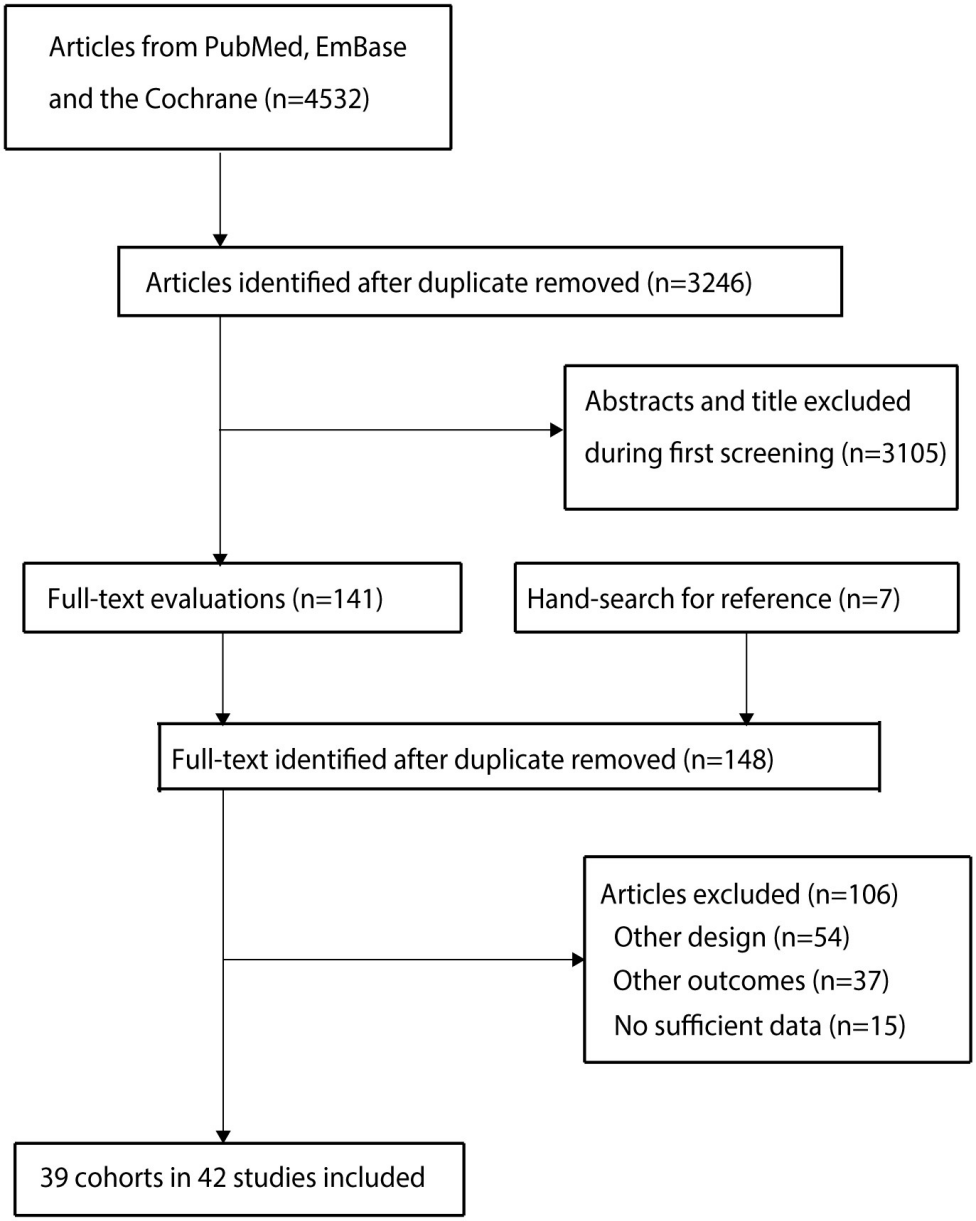
The diagram for literature search and study selection process.

An initial electronic searches yields 4,532 records, and 3,246 articles were retained after duplicate records were excluded. Then a total of 3,105 studies were removed owing to these studies reported irrelevant topics. Reviewing the reference lists yields 7 potential included studies, and a total of 148 studies were retrieved for full-text evaluations. After this, 106 studies were excluded because of: other design (n = 54), reported other outcomes (n = 37), no sufficient data (n = 15). Finally, 39 cohorts reported in 42 studies were selected for meta-analysis [26–67]. Fig 1 presented the details of literature search and study selection.

### Characteristics of the included studies

The baseline characteristics of included studies and involved individuals are shown in Table 1. A total of 4,389,263 individuals were included, and the sample size in each study ranged from 117 to 975,685. Ten cohorts were performed in Eastern countries (China, Korea, Japan, and Thailand), while the remaining 29 cohorts were conducted in Western countries (USA, UK, Canada, Denmark, Sweden, Finland, Australia, and Poland). Twenty-nine studies designed as prospective cohort, while the remaining 10 studies designed as retrospective cohort. Ten studies used self-report to defined PD, while the remaining 29 studies applied clinical diagnosed for PD definition. The follow-up duration of included studies ranged from 2.9 years to 57.0 years, and the study quality with 6–8 stars.

**Table 1:** The baseline characteristics of included studies and involved individuals.

Study	Region	Study design	Sample size	Age (years)	Male (%)	PD definition	Reported outcomes	Follow-up duration	Adjusted factors	NOS scale
DeStefano 1993 [26]	USA	Pro	9,760	25–74	38.8	Clinical	CHD, all-cause mortality	16.0 years	Age, sex, race, education, poverty index, marital state, SBP, TC, DM, BMI, PA, alcohol, and smoking	7
Beck 1996 [27]	USA	Pro	1,147	NA	NA	Clinical	CHD, cardiac death, stroke	18.0 years	Age, smoking, DM, DBP, family history, education	6
Joshipura 1996 [28]	USA	Pro	44,119	40–75	100.0	Self-report	CHD	6.0 years	Age, BMI, PA, smoking, alcohol, family history of MI, and vitamin E	8
Morrison 1999 [29]	Canada	Retro	9,331	35–84	45.5	Clinical	Cardiac death, stroke	23.0 years	Age, sex, TC, smoking, DM, hypertension, and province of residence	6
Wu 2000 [30]	USA	Pro	9,962	25–74	38.0	Clinical	Stroke	22.0 years	Sex, race, age, education, poverty index, DM, hypertension, smoking, alcohol, BMI, and TC	7
Howell 2001 [31]	USA	Pro	22,037	40–84	100.0	Self-report	MACE, MI, cardiac death, stroke	12.3 years	Age, aspirin and beta-carotene treatment, smoking, alcohol, hypertension, BMI, DM, PA, parental history of MI and history of angina	8
Hujoel 2001 [32]	USA	Pro	4,027	55–74	44.8	Clinical	CHD, Cardiac death	17.0 years	BMI, PA, nervous breakdown, smoking, DBP, SBP, TC, and DM	6
Elter 2003 [33]	USA	Retro	10,906	NA	NA	Clinical	Stroke	9.0 years	Age, race, education, hypertension, smoking, DM, CHD, LDL, HDL, TG, BMI	7
Joshipura 2003 [34]	USA	Pro	41,380	40–75	100.0	Self-report	Stroke	12.0 years	Age, smoking, alcohol, BMI, PA, family history of MI, multivitamin, vitamin E, hypertension, DM, hypercholesterolemia, and professions	8
Hung 2004 [35]	USA	Pro	100,381	30–75	41.2	Self-report	CHD	12.0 years	Age, smoking, alcohol, BMI, PA, family history of MI, multivitamin, vitamin E, hypertension, DM, and hypercholesterolemia, professions for men only, and for women only, menopausal status and hormone	8
Abnet 2005 [36]	China	Pro	29,584	40–69	45.0	Clinical	MACE, stroke, all-cause mortality	15.0 years	Age, sex, smoking, height, weight, and SBP	7
Tu 2007 [37]	UK	Pro	12,631	16–30	78.3	Clinical	MACE, CHD, stroke	57.0 years	Age, year of birth, gender, smoking, BMI, SBP, father’s socioeconomic status	7
Dietrich 2008 [38]	USA	Pro	1,203	21–84	100.0	Clinical	CHD, Cardiac death	24.0 years	Age, BMI, HDL, TC, TG, hypertension, SBP, DBP, DM, fasting glucose, smoking, alcohol, occupation and education, income, and marital status	7
Heitmann 2008 [39]	Denmark	Pro	2,932	NA	50.3	Clinical	MACE, CHD, stroke	7.0 years	Age and whether number of teeth was counted in 1987–88 or 1993–94	6
Choe 2009 [40]	Korea	Pro	867,256	> 30	78.3	Clinical	Stroke	14.0 years	Age, obesity, hypercholesterolemia, hypertension, DM, alcohol, PA, and smoking	7
Jimenez 2009 [41]	USA	Pro	1,137	27–84	100.0	Clinical	Stroke	34.0 years	Age, BMI, HDL, TC, TG, hypertension, SBP, DBP, DM, alcohol, smoking, marital status, and baseline measures of education, occupation, and income	7
Mucci 2009 [42]	Sweden	Pro	15,273	35	43.9	Self-report	MACE, CHD, stroke	37.0 years	Age, sex, early-life exposures, familial factors, number of siblings, smoking, DM, hypertension, and BMI	7
Dorn 2010 [43]	USA	Pro	884	35–69	75.6	Clinical	MACE	2.9 years	Age, gender, education, DM, TC, lipid-lowering medication, hypertension medication, BMI, recreational, PA, and fruit and vegetable intake	8
Lee 2013 [44,45]	China	Retro	719,436	> 20	51.1	Clinical	Stroke, MI	10.0 years	Age, sex, hypertension, DM, atrial fibrillation, atherosclerosis, chronic kidney disease, dyslipidemia	7
Lijestrand 2015 [46]	Finland	Retro	7,629	25–74	NA	Clinical	NACE, CHD, MI, stroke, all-cause mortality	13.0 years	SBP, anti-hypertensive drugs, TC, HDL, education, and DM	7
Chou 2015 [47]	China	Retro	27,146	> 18	52.6	Clinical	MACE	9.0 years	Sex, hyperlipidemia, hypertension, DM, and treated periodontitis stage	6
Noguchi 2015 [48]	Japan	Pro	3,081	36–59	100.0	Self-report	MI	5.0 years	Age, BMI, smoking, hypertension, DM, dyslipidemia, family history of CVD	6
Yu 2015 [49]	USA	Pro	39,863	> 45	0.0	Self-report	MACE, MI, stroke	15.7 years	Age, race/ethnicity, BMI, education, smoking, DM, hypertension, hypercholesterolemia, family history of MI, PA, CRP	8
Hansen 2016 [50]	Denmark	Pro	100,694	> 18	57.0	Clinical	MACE, MI, stroke, cardiac death, all-cause mortality	15.0 years	Age, sex, smoking, comorbidities, medication, and socio-economic status	8
Joshy 2016 [51]	Australia	Pro	172,630	45–75	43.0	Self-report	CHD, stroke, all-cause mortality	5.0 years	Age, sex, smoking, alcohol, Australian born status, region of residence, education, health insurance, PA and BMI	7
Holmlund 2017 [52]	Sweden	Pro	8,999	20–85	43.0	Clinical	MACE, MI, stroke	15.8 years	Age, sex, education level and smoking	7
LaMonte 2017 [53]	USA	Pro	57,001	55–89	0.0	Self-report	MACE, CHD, stroke, cardiac death, all-cause mortality	6.7 years	Age, smoking, dental visits, DM, race-ethnicity, education, hypertension, TC, BMI, PA, alcohol, and dietary healthy eating index	8
Lee 2017 [54,55]	Korea	Retro	354,850	40–79	56.3	Clinical	MI, stroke, all-cause mortality	12.0 years	Sex, age, household income, insurance status, residence area, health status, and smoking	6
Batty 2018 [56]	Korea	Pro	975,685	30–95	64.2	Clinical	CHD	21.0 years	Age, socioeconomic status, height, alcohol, smoking, PA, SBP, TC, DM, BMI, family history of CVD	7
Sen 2018 [57,58]	USA	Retro	6,736	45–64	45.0	Clinical	Stroke, CHD, MI, cardiac death, all-cause morality	15.0 years	Race/center, age, sex, BMI, hypertension, DM, LDL, smoking, education	6
Lin 2019 [59]	China	Retro	161,925	> 20	54.2	Clinical	Stroke	10.0 years	Sex, age, and comorbidities	7
Khouja 2019 [60]	USA	Pro	320	32	55.6	Clinical	CHD	19.0 years	Age, duration of DM, education, and smoking	7
Wynimko 2020 [61]	Poland	Pro	117	44	65.8	Clinical	MACE, all-cause mortality	15.0 years	Age, sex, dialysis vintage, delayed graft function, smoking, CRP, serum creatinine, SBP, DBP	6
Qi 2020 [62]	China	Retro	1,385	> 75	47.9	Clinical	Cardiac death, all-cause mortality	8.0 years	Age, sex, education levels, BMI, smoking, drinking, hypertension, and DM	6
Kotronia 2021 [63]	UK and USA	Pro	5,145	70–92	70.7	Clinical	Cardiac death, all-cause mortality	9.0 and 15.0 years	Age, social class, smoking, alcohol, PA, history of CVD and DM, BMI, EDI score, hypertension, TG, HDL, CRP, IL-6,fibrin d-dimer, hsTnT.	7
Zemedikun 2021 [64]	UK	Retro	315,540	> 18	43.0	Clinical	MACE, CHD, stroke	3.4 years	Age, sex, BMI, Townsend Deprivation Index, smoking and ethnicity at baseline	7
Tiensripojamarn 2021 [65]	Thailand	Pro	1,850	47–73	74.0	Clinical	MACE, CHD, stroke	13.0 years	Age, sex, smoking, alcohol, hypertension, DM, TC, HDL, LDL, and waist circumference	7
Larvin 2022 [66]	UK	Pro	244,393	49–60	46.7	Self-report	MACE, all-cause mortality	6.4 years	Age, sex, BMI, ethnicity, household income, CRP, smoking, and hypertension	8
Hamaya 2023 [67]	USA	Pro	888	64	27.3	Clinical	MACE	4.6 years	Age, sex, BMI, alcohol, smoking, disease leading to the hospital admission, DM, hypertension, hypercholesterolemia, left ventricular ejection fraction, aspirin and statin use	6

*BMI: Body mass index; CHD: Coronary heart disease; CRP: C-reactive protein; CVD: Cardiovascular disease; DBP: Diastolic blood pressure; DM: Diabetes mellitus; HDL: High-density lipoprotein; LDL: Low-density lipoprotein; LVEF: Left ventricular ejection fraction; MACE: Major cardiovascular events; MI: Myocardial infraction; MI: Myocardial infraction; PA: activity; Pro: Prospective; Retro: Retrospective; SBP: Systolic blood pressure; TC: Total cholesterol; TG: Triglycerides.

### Major adverse cardiovascular events

**Fig 2:**
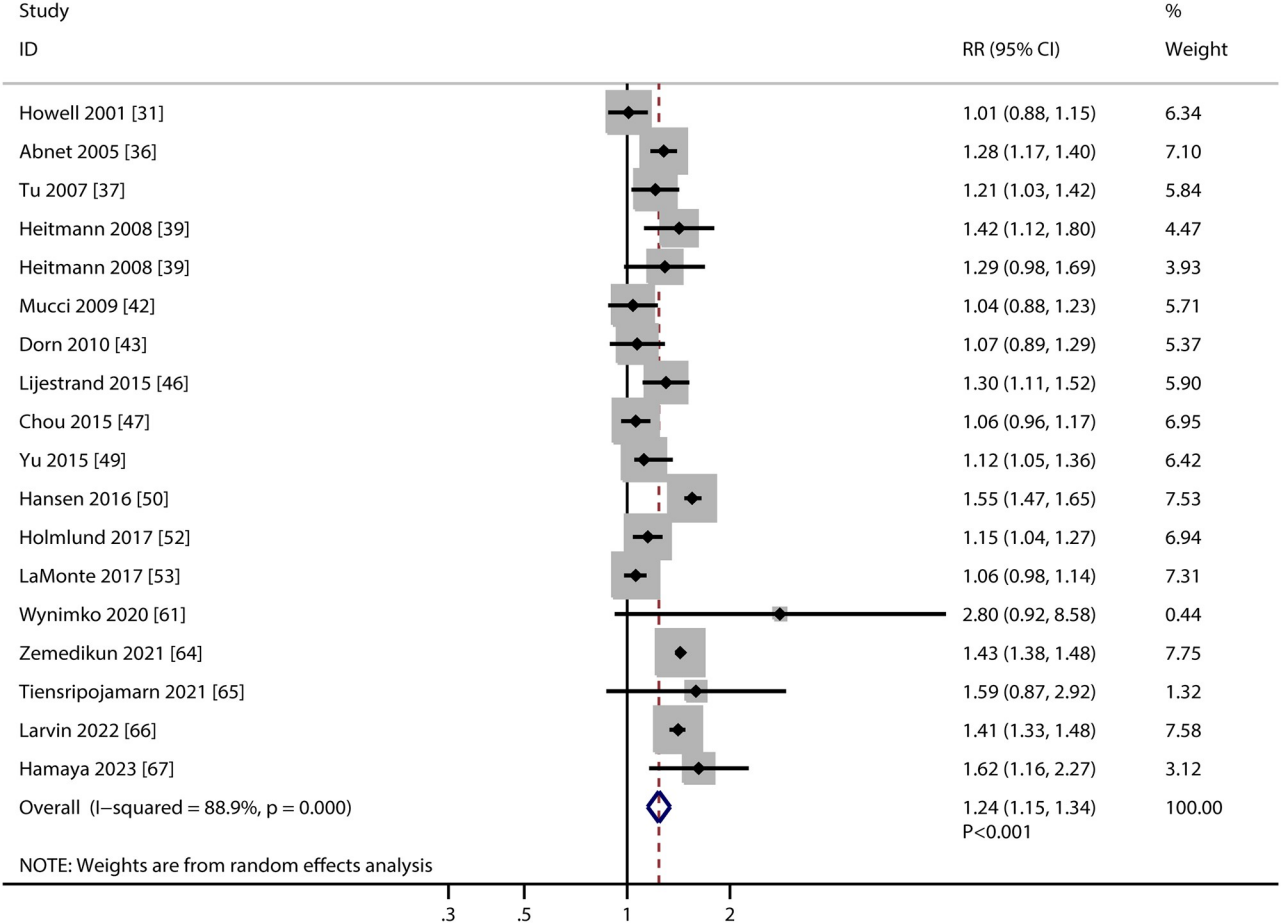
Association of periodontal disease with the risk of major adverse cardiovascular events.

The association of PD with the risk of MACEs were available in 17 studies, and the summary RR indicated PD was associated with an increased risk of MACEs (RR: 1.24; 95%CI: 1.15–1.34; *P*<0.001; Fig 2). The heterogeneity across included studies was substantial (*I*^*2*^ = 88.9%; *P*<0.001). Sensitivity analysis was performed to assess the role of single study from the overall analysis, and the results found the pooled conclusion was stability and the heterogeneity was not significantly reduced (S1 File). Subgroup analysis indicated elevated MACEs risk in patients with PD in mostly subgroups, whereas PD was not associated with the risk of MACEs if pooled male individuals, and used self-report as PD definition (Table 2). There was no significant publication bias for the association of PD with the risk of MACEs (*P* value for Egger: 0.095; *P* value for Begg: 0.325; S2 File).

**Table 2:** Subgroup analyses for cardiovascular outcomes and all-cause mortality.

Outcomes	Factors	Subgroups	No of studies	RR and 95%CI	*P* value	I^2^ (%)	Q statistic	Interaction *P* value	RRR between subgroups
MACE	Region	Eastern	3	1.19 (1.00–1.42)	0.048	76.8	0.014	0.617	0.95 (0.79–1.15)
		Western	14	1.25 (1.15–1.35)	< 0.001	89.3	< 0.001		
	Study design	Prospective	14	1.24 (1.13–1.36)	< 0.001	87.7	<0.001	0.891	0.98 (0.78–1.24)
		Retrospective	3	1.26 (1.02–1.55)	0.032	93.7	< 0.001		
	Gender	Male	3	1.27 (0.98–1.64)	0.070	88.0	< 0.001	0.255	1.17 (0.90–1.52)
		Female	4	1.09 (1.03–1.15)	0.004	0.0	0.537		
	PD definition	Clinical	12	1.30 (1.19–1.41)	< 0.001	84.1	< 0.001	0.128	1.15 (0.96–1.38)
		Self-report	5	1.13 (0.96–1.32)	0.147	92.6	< 0.001		
	Follow-up (years)	≥ 10.0	10	1.22 (1.09–1.38)	0.001	87.1	< 0.001	0.701	0.97 (0.82–1.14)
		< 10.0	7	1.26 (1.12–1.41)	< 0.001	91.5	< 0.001		
	Adjusted level	High	10	1.18 (1.05–1.34)	0.006	85.4	< 0.001	0.221	0.91 (0.78–1.06)
		Moderate	7	1.30 (1.18–1.43)	< 0.001	88.9	< 0.001		
	Study quality	High	13	1.23 (1.13–1.33)	< 0.001	90.7	< 0.001	0.503	0.92 (0.74–1.16)
		Moderate	4	1.33 (1.07–1.64)	0.009	68.6	0.013		
CHD	Region	Eastern	2	1.04 (0.97–1.10)	0.274	70.3	0.035	< 0.001	0.85 (0.78–0.93)
		Western	15	1.22 (1.15–1.30)	< 0.001	66.6	< 0.001		
	Study design	Prospective	14	1.16 (1.09–1.22)	< 0.001	74.9	< 0.001	0.026	0.86 (0.75–0.98)
		Retrospective	3	1.35 (1.20–1.53)	< 0.001	59.3	0.086		
	Gender	Male	7	1.17 (1.06–1.30)	0.003	77.4	< 0.001	0.588	1.04 (0.89–1.22)
		Female	5	1.12 (0.99–1.26)	0.065	77.3	0.001		
	PD definition	Clinical	12	1.21 (1.11–1.33)	< 0.001	92.6	< 0.001	0.578	1.03 (0.92–1.16)
		Self-report	5	1.17 (1.09–1.27)	< 0.001	50.7	0.058		
	Follow-up (years)	≥ 10.0	12	1.21 (1.13–1.30)	< 0.001	83.3	< 0.001	0.477	1.05 (0.91–1.21)
		< 10.0	5	1.15 (1.02–1.30)	0.022	81.2	< 0.001		
	Adjusted level	High	10	1.16 (1.08–1.23)	< 0.001	79.5	< 0.001	0.276	0.94 (0.83–1.05)
		Moderate	7	1.24 (1.12–1.37)	< 0.001	71.5	< 0.001		
	Study quality	High	13	1.21 (1.12–1.30)	< 0.001	91.9	< 0.001	0.743	1.03 (0.85–1.26)
		Moderate	4	1.17 (0.97–1.41)	0.107	58.1	0.049		
MI	Region	Eastern	3	1.07 (0.97–1.17)	0.182	90.0	< 0.001	0.120	0.88 (0.76–1.03)
		Western	6	1.21 (1.07–1.37)	0.003	64.5	0.015		
	Study design	Prospective	5	1.15 (1.05–1.26)	0.003	19.8	0.288	0.801	1.02 (0.89–1.17)
		Retrospective	4	1.13 (1.02–1.25)	0.022	90.8	< 0.001		
	Gender	Male	2	1.30 (0.63–2.71)	0.482	59.4	0.117	0.924	0.96 (0.45–2.08)
		Female	1	1.35 (1.05–1.73)	0.018	-	-		
	PD definition	Clinical	6	1.13 (1.04–1.22)	0.003	85.6	< 0.001	0.626	0.93 (0.68–1.26)
		Self-report	3	1.22 (0.91–1.65)	0.186	59.2	0.086		
	Follow-up (years)	≥ 10.0	8	1.13 (1.05–1.22)	0.001	81.6	< 0.001	0.170	0.50 (0.19–1.35)
		< 10.0	1	2.26 (0.84–6.05)	0.105	-	-		
	Adjusted level	High	5	1.09 (0.97–1.24)	0.156	53.4	0.072	0.274	0.91 (0.76–1.08)
		Moderate	4	1.20 (1.06–1.35)	0.003	77.2	0.004		
	Study quality	High	6	1.18 (1.03–1.34)	0.014	82.2	< 0.001	0.372	1.06 (0.93–1.22)
		Moderate	3	1.11 (1.08–1.14)	< 0.001	0.4	0.366		
Stroke	Region	Eastern	6	1.10 (0.96–1.27)	0.176	97.7	< 0.001	0.025	0.82 (0.69–0.98)
		Western	18	1.34 (1.21–1.48)	< 0.001	80.9	< 0.001		
	Study design	Prospective	16	1.26 (1.14–1.40)	< 0.001	78.4	< 0.001	0.862	1.02 (0.85–1.22)
		Retrospective	8	1.24 (1.07–1.44)	0.005	98.0	< 0.001		
	Gender	Male	6	1.35 (1.15–1.59)	< 0.001	60.5	0.027	0.961	1.01 (0.75–1.36)
		Female	5	1.34 (1.04–1.72)	0.023	84.0	< 0.001		
	PD definition	Clinical	18	1.31 (1.19–1.46)	< 0.001	96.0	< 0.001	0.012	1.19 (1.04–1.37)
		Self-report	6	1.10 (1.00–1.20)	0.045	0.0	0.550		
	Follow-up (years)	≥ 10.0	19	1.22 (1.11–1.35)	< 0.001	95.1	< 0.001	0.274	0.90 (0.74–1.09)
		< 10.0	5	1.36 (1.15–1.61)	< 0.001	83.1	< 0.001		
	Adjusted level	High	14	1.22 (1.05–1.42)	0.009	93.5	< 0.001	0.662	0.96 (0.80–1.15)
		Moderate	10	1.27 (1.15–1.40)	< 0.001	88.8	< 0.001		
	Study quality	High	19	1.16 (1.04–1.30)	0.008	94.6	< 0.001	0.010	0.59 (0.39–0.88)
		Moderate	5	1.98 (1.34–2.92)	0.001	92.4	< 0.001		
Cardiac death	Region	Eastern	1	0.89 (0.65–1.22)	0.468	-	-	0.012	0.59 (0.39–0.89)
		Western	9	1.50 (1.15–1.94)	0.002	91.0	<0.001		
	Study design	Prospective	7	1.41 (1.03–1.93)	0.031	93.2	< 0.001	0.895	0.96 (0.52–1.78)
		Retrospective	3	1.47 (0.86–2.50)	0.159	84.4	0.002		
	Gender	Male	4	1.36 (1.05–1.78)	0.020	68.3	0.024	0.244	1.19 (0.89–1.61)
		Female	2	1.14 (0.99–1.30)	0.068	0.0	0.331		
	PD definition	Clinical	8	1.55 (1.18–2.04)	0.001	86.7	< 0.001	0.014	1.46 (1.08–1.98)
		Self-report	2	1.06 (0.93–1.21)	0.383	0.0	0.550		
	Follow-up (years)	≥ 10.0	8	1.57 (1.20–2.05)	0.001	87.9	< 0.001	0.010	1.52 (1.11–2.10)
		< 10.0	2	1.03 (0.87–1.23)	0.721	21.1	0.260		
	Adjusted level	High	7	1.35 (1.09–1.67)	0.006	74.3	0.001	0.742	0.90 (0.48–1.69)
		Moderate	3	1.50 (0.83–2.70)	0.177	91.9	< 0.001		
	Study quality	High	5	1.45 (1.00–2.09)	0.051	94.3	< 0.001	0.869	1.04 (0.63–1.72)
		Moderate	5	1.39 (0.99–1.95)	0.060	78.9	0.001		
All-cause mortality	Region	Eastern	3	1.19 (1.09–1.30)	< 0.001	89.9	< 0.001	0.387	0.86 (0.62–1.21)
		Western	9	1.38 (1.00–1.91)	0.052	99.2	< 0.001		
	Study design	Prospective	8	1.41 (1.01–1.97)	0.046	99.4	< 0.001	0.377	1.17 (0.83–1.64)
		Retrospective	4	1.21 (1.14–1.28)	< 0.001	38.1	0.183		
	Gender	Male	3	1.36 (1.07–1.74)	0.013	73.1	0.024	0.080	1.25 (0.97–1.60)
		Female	2	1.09 (1.04–1.14)	< 0.001	0.0	0.337		
	PD definition	Clinical	9	1.38 (1.06–1.79)	0.017	99.4	< 0.001	0.344	1.18 (0.84–1.66)
		Self-report	3	1.17 (0.94–1.46)	0.165	96.0	< 0.001		
	Follow-up (years)	≥ 10.0	8	1.40 (1.06–1.86)	0.018	99.5	< 0.001	0.294	1.20 (0.86–1.67)
		< 10.0	4	1.17 (0.98–1.41)	0.088	94.0	< 0.001		
	Adjusted level	High	6	1.15 (1.01–1.31)	0.032	87.5	< 0.001	0.243	0.82 (0.58–1.15)
		Moderate	6	1.41 (1.03–1.94)	0.032	99.6	< 0.001		
	Study quality	High	8	1.31 (0.96–1.81)	0.093	99.4	< 0.001	0.640	1.08 (0.78–1.51)
		Moderate	4	1.21 (1.09–1.33)	< 0.001	62.8	0.045		

### Coronary heart disease

**Fig 3:**
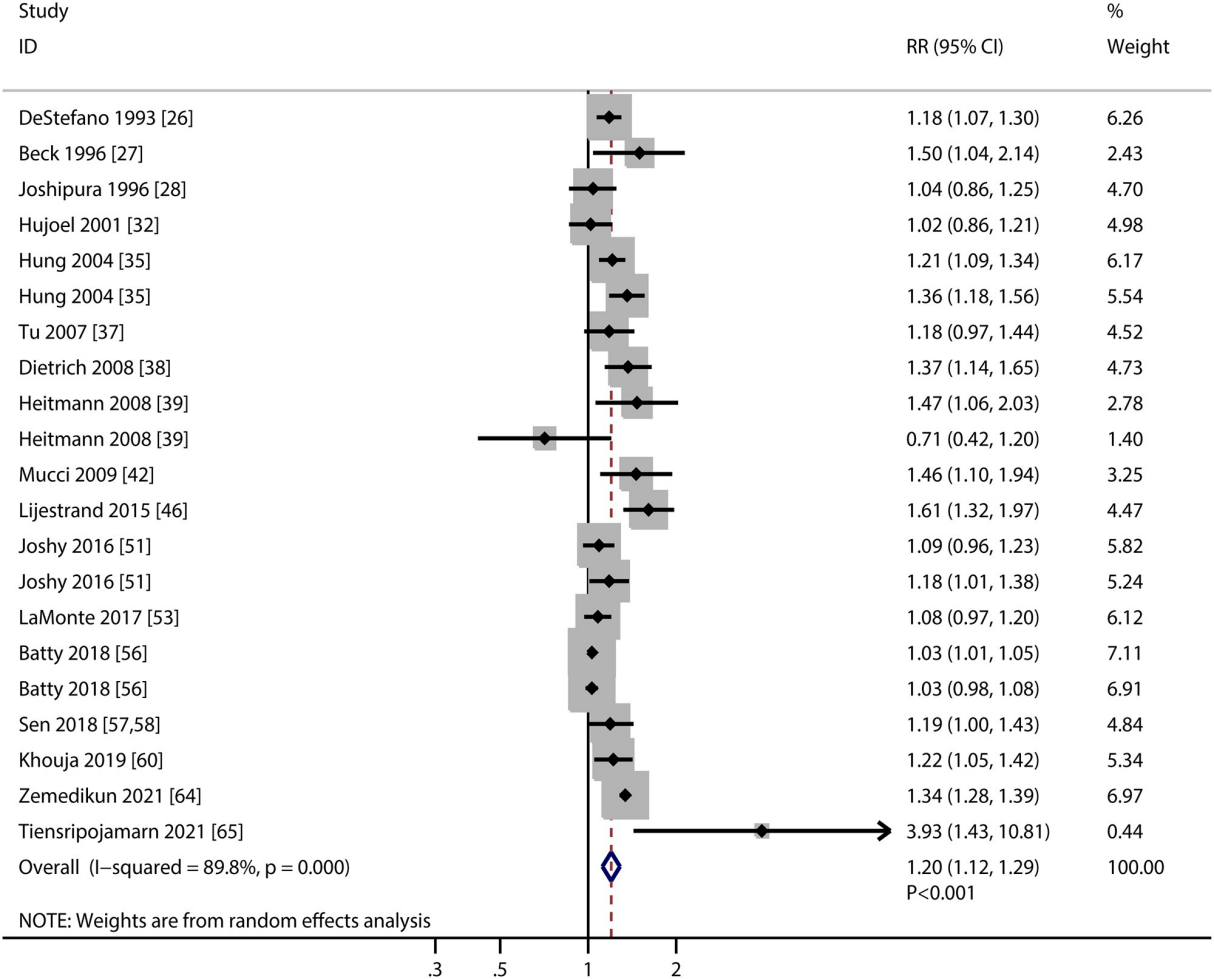
Association of periodontal disease with the risk of coronary heart disease.

A total of 17 studies investigated the association of PD with the risk of CHD. We noted PD was associated with an elevated risk of CHD (RR: 1.20; 95%CI: 1.12–1.29; *P*<0.001; Fig 3), and significant heterogeneity among included studies was observed (*I*^*2*^ = 89.8%; *P*<0.001). The pooled conclusion was robustness and not altered by excluding any particular study. Moreover, the heterogeneity was not significantly changed by removing any individual study (S1 File). The significant associations between PD and CHD were observed in mostly subgroups, whereas PD did not affected subsequent risk of CHD if pooled studies conducted in Eastern countries, female individuals, and studies with moderate quality (Table 2). The effect estimates for the relationship between PD and CHD in the subgroups of Western countries (*P*<0.001), and retrospective cohort studies (*P* = 0.026) were higher than corresponding subgroups. Although the Begg test indicated no significant publication bias (*P* = 0.415), while the Egger test suggested potential significant publication bias for CHD (*P* = 0.029) (S2 File). The conclusions were not changed after adjustment for publication bias by using the trim and fill method [68].

### Myocardial infarction

**Fig 4:**
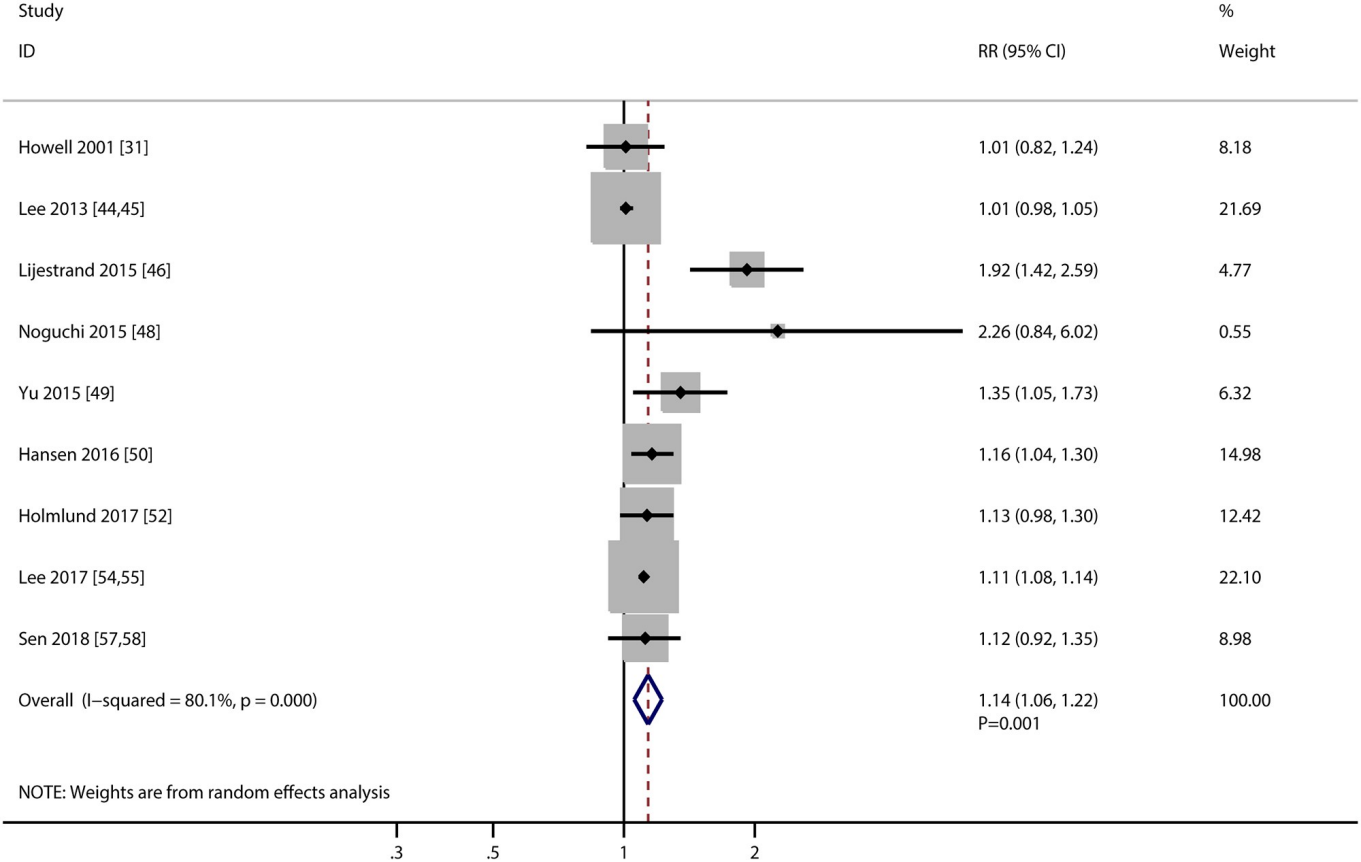
Association of periodontal disease with the risk of myocardial infraction.

The association of PD with the risk of MI were available in 9 studies, and the summary result indicated PD was associated with an increased risk of MI (RR: 1.14; 95%CI: 1.06–1.22; *P* = 0.001; Fig 4). There was significant heterogeneity among included studies (*I*^*2*^ = 80.1%; *P*<0.001), and this significant heterogeneity was not significantly altered by removing any particular study. Moreover, the pooled conclusion was stability and not changed by removing any single study (S1 File). Although PD was associated with the risk of MI in mostly subgroups, whereas PD was not associated with the risk of MI if pooled studies conducted in Eastern countries, male individuals, used self-report defined PD, follow-up duration <10.0 years, and studies with high adjusted level (Table 2). No significant publication bias MI were observed (*P* value for Egger: 0.181; *P* value for Begg: 0.602; S2 File).

### Stroke

**Fig 5:**
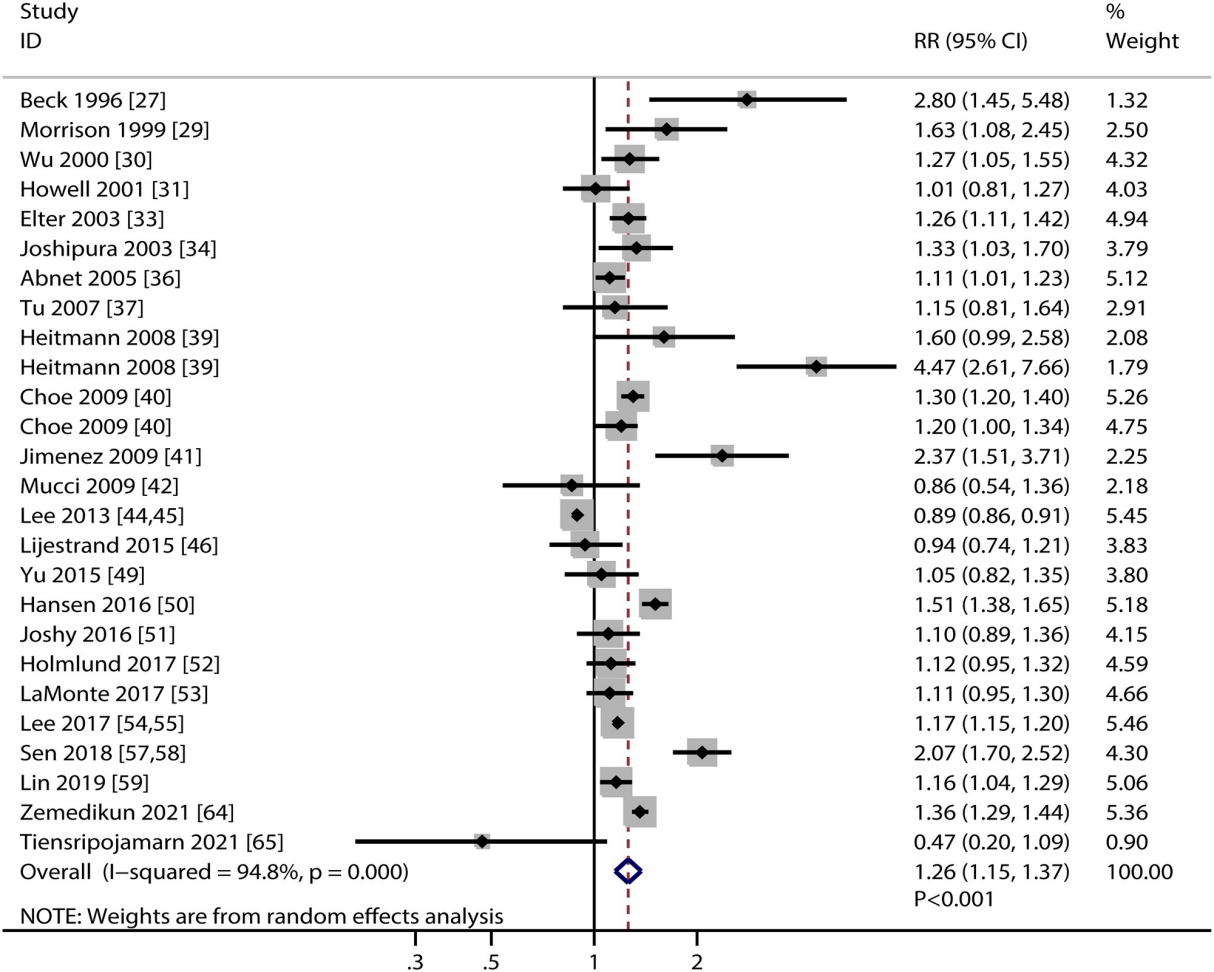
Association of periodontal disease with the risk of stroke.

A total of 24 studies investigated the association of PD with the risk of stroke. We noted PD was associated with an increased risk of stroke (RR: 1.26; 95%CI: 1.15–1.37; *P*<0.001; Fig 5), and significant heterogeneity was observed across included studies (*I*^*2*^ = 94.8%; *P*<0.001). Sensitivity analysis indicated the pooled conclusion was stability and the heterogeneity was not significantly changed by sequential removing individual study (S1 File). Subgroup analysis found significant association between PD and stroke in mostly subgroups, whereas PD was not associated with the risk of stroke if pooled studies conducted in Eastern countries (Table 2). Region (*P* = 0.025), PD definition (*P* = 0.012), and study quality (*P* = 0.010) could affect the association between PD and stroke risk. There was no significant publication bias for the association of PD with the risk of stroke (*P* value for Egger: 0.109; *P* value for Begg: 0.355; S2 File).

### Cardiac death

**Fig 6:**
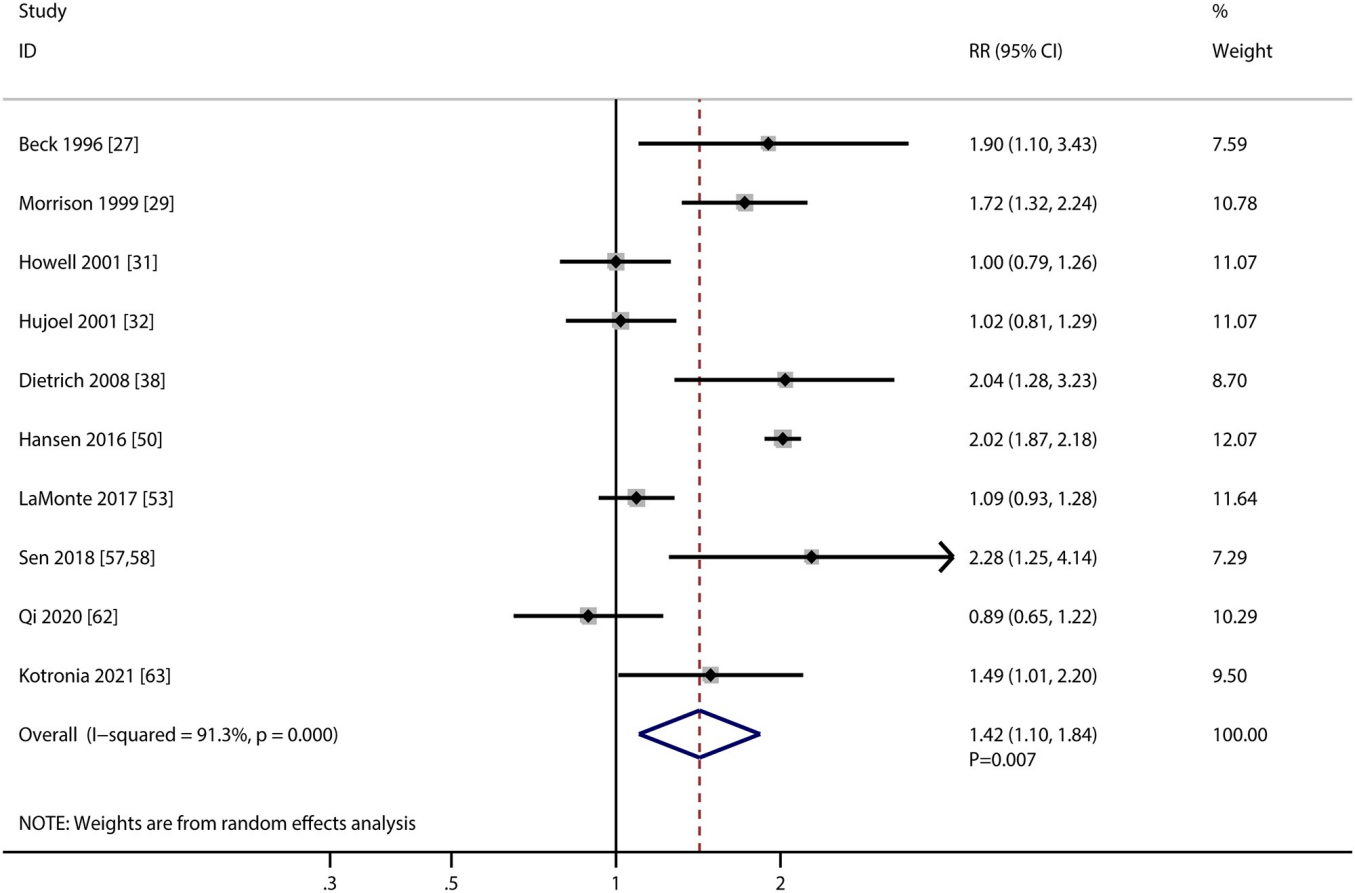
Association of periodontal disease with the risk of cardiac death.

The association of PD with the risk of cardiac death were available in 10 studies, and the summary RR indicated PD was associated with an increased risk of cardiac death (RR: 1.42; 95%CI: 1.10–1.84; *P* = 0.007; Fig 6). There was significant heterogeneity across included studies (*I*^*2*^ = 91.3%; *P*<0.001). The pooled conclusion and heterogeneity among included studies were not significantly altered by sequential removing individual study (S1 File). Subgroup analyses found PD was associated with an increased risk of cardiac death when pooled studies conducted in Western countries, studies designed as prospective cohort, male individuals, used clinical diagnosed defined PD, follow-up duration ≥ 10.0 years, and studies with high adjusted level (Table 2). The association of PD and the risk of cardiac death could affected by region (*P* = 0.012), PD definition (*P* = 0.014), and follow-up duration (*P* = 0.010). No significant publication bias for cardiac death was observed (*P* value for Egger: 0.194; *P* value for Begg: 0.107; S2 File).

### All-cause mortality

**Fig 7:**
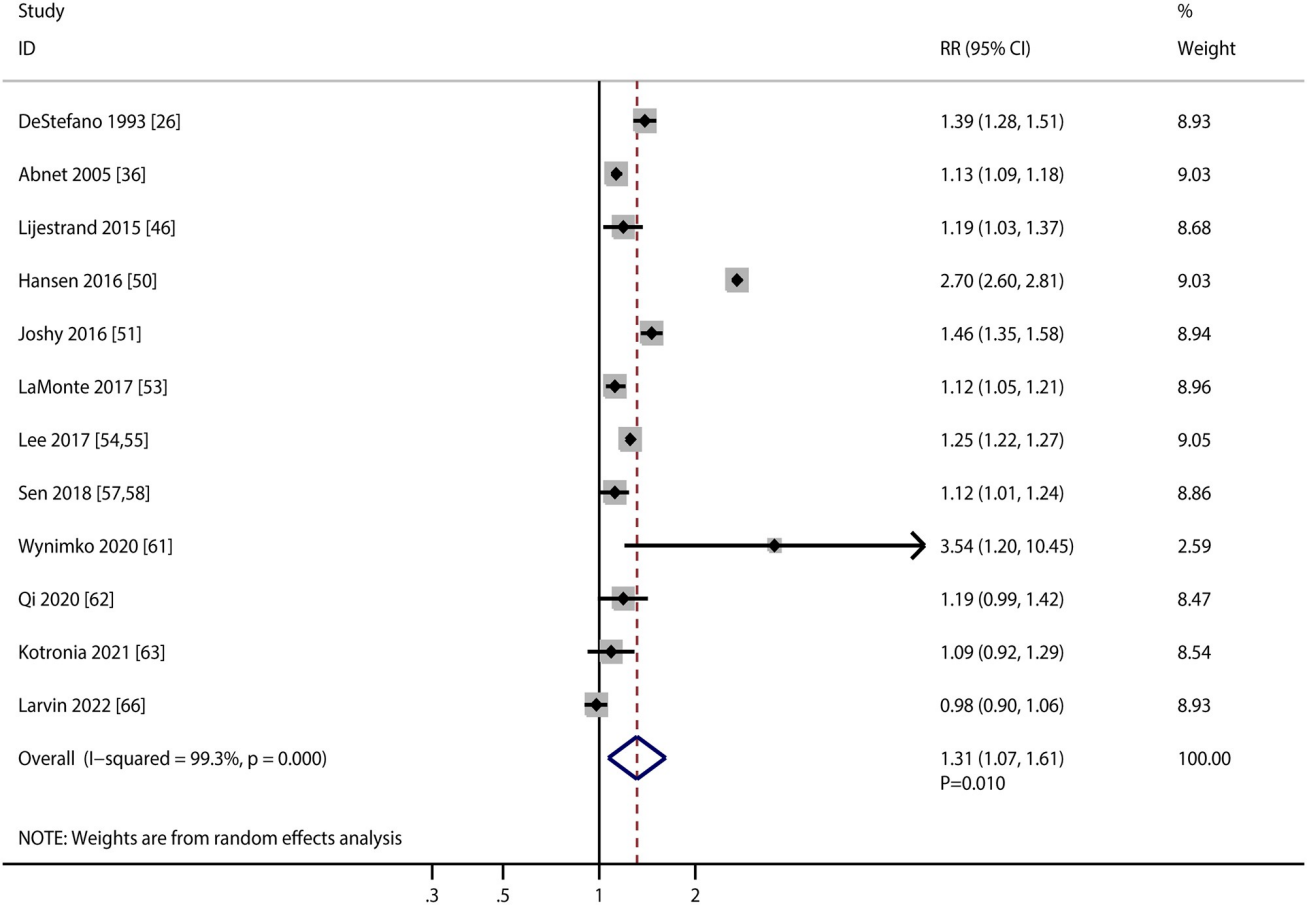
Association of periodontal disease with the risk of all-cause mortality.

A total of 12 studies investigated the association of PD with the risk of all-cause mortality. We noted PD was associated with an increased risk of all-cause mortality (RR: 1.31; 95%CI: 1.07–1.61; *P* = 0.010; Fig 7), and significant heterogeneity was detected among included studies (*I*^*2*^ = 99.3%; *P*<0.001). Sensitivity analysis indicated the pooled conclusion was not altered and the heterogeneity was not fully explained by excluding any specific study (S1 File). Subgroup analysis found PD was associated with an elevated risk of all-cause mortality in mostly subgroups, whereas PD did not affected subsequent all-cause mortality risk if pooled studies conducted in Western countries, used self-report defined PD, follow-up duration < 10.0 years, and studies with high quality (Table 2). There was no significant publication bias for all-cause mortality (*P* value for Egger: 0.914; *P* value for Begg: 0.150; S2 File).

## Discussion

The current systematic review and meta-analysis on the basis of cohort studies and investigated the associations of PD with the risk of MACEs, CHD, MI, stroke, cardiac death, and all-cause mortality. This comprehensive, quantitative analysis identified 4,389,263 individuals from 29 prospective cohort studies and 10 retrospective cohort studies across wide range of individuals’ characteristics. Our study found PD were associated with elevated risk of MACEs, CHD, MI, stroke, cardiac death, and all-cause mortality. Moreover, the association of PD with the risk of CHD in the subgroups of studies conducted in Western countries, and studies designed as retrospective cohort were greater than corresponding subgroups. Furthermore, PD and subsequent risk of stroke in the subgroups of studies conducted in Western countries, PD with clinical diagnosed, and studies with moderate quality were greater than corresponding subgroups. Finally, the effect estimates for the relation between PD and cardiac death in the subgroups of Western countries, PD with clinical diagnosed, and follow-up duration ≥ 10.0 years were larger than corresponding subgroups.

Several systematic review and meta-analyses have already investigated the role of PD with the risk of cardiovascular outcomes and all-cause mortality [13–15]. Larvin et al identified 30 cohort studies and found the MACEs risk was significantly elevated in PD patients, and the risk increased highest was stroke [13]. Qin et al performed a meta-analysis of 10 cohort studies and found PD was associated with an increased risk of MI, while this association could affected by sex, effect value, survey form, and study quality [14]. Leng et al identified 26 studies and found significant association between PD and MACEs in both men and women [15]. However, several other important outcomes, including cardiac death, and all-cause mortality were not investigated. Moreover, whether the associations of PD with the risk of cardiovascular outcomes and all-cause mortality could affected by individuals’ characteristics were not fully illustrated. Therefore, the current study was performed to assess the role of PD with subsequent cardiovascular outcomes and all-cause mortality risk.

This study found PD were associated with elevated risk of MACEs, CHD, MI, stroke, cardiac death, and all-cause mortality. The potential mechanism for these relationships included: (1) The PD pathogens entering the blood can directly invade the cardiovascular endothelial cells and smooth muscle cells, which significantly associated with the risk of vascular diseases. Moreover, the pathogens could colonize at atherosclerotic plaque, pericarditis or myocardial tissue after enter blood, which could affect the occurrence and development of coronary heart disease, occurrence and development of coronary heart disease [69–71]; (2) Porphyromonas gingivalis, as one of the main pathogens of PD, it can induce the expression of platelet aggregation and intercellular adhesion molecule-1, vascular cell adhesion molecule-1 and P-selectin. In addition, the arginine-specific cysteine proteinase R released by it can induce CVD by activating protein C, coagulation factor X and prothrombin [72]; (3) PD pathogens could induce systemic inflammation, which demonstrated inflammation was considered as a predisposing factor for CVD [73,74]. PD patients were associated with elevated levels of C-reactive protein and other inflammatory markers in the circulation, which could promote the progression of systemic inflammation and CVD [75,76]; (4) Oral pathogens could cross the gingival epithelial-conjunctival barrier and vascular endothelial cells, then reached the blood, causing the inflammation and immune response in blood vessels [77–80]; (5) PD pathogens could induce myocardial hypertrophy via activated oxidative stress pathway [81]; and (6) PD was associated with a reduced vascular endothelial function in the gingival tissue, and damage of vascular function, then caused excess risk of stroke [82].

Subgroup analyses found the associations of PD with the risk of CHD, stroke, and cardiac death could affected by region, study design, PD definition, follow-up duration, and study quality. Several reasons could explained these results: (1) the excess risk of CHD, stroke, and cardiac death related to PD in Western countries might caused by older age and severity of PD. Moreover, large number of included studies were performed in Western countries, and the conclusions was robust with narrow 95%CI; (2) although effect estimate for the relation between PD and CHD in retrospective cohort studies was large than prospective cohort studies, while these results was variable owing to only 3 included studies designed as retrospective cohort. Moreover, prospective cohort studies could eliminate selection and recall bias that might be concerned of retrospective cohort studies; (3) the definition of PD applied clinical diagnosis was more accuracy than self-report, and the severity of PD could affect the effect estimates of cardiovascular outcomes [13]; (4) considering the progression of cardiac death related to PD are slowing, longer follow-up duration could observed large number of cardiac death, thus the power was enough to detect potential association; and (5) the methodological quality were assessed based on selection, comparability, and outcome, which is significantly related to the reliable of pooled conclusion. Moreover, smaller number of studies with moderate quality, and the results not stability.

Several strengths of this study should be mentioned. First, the analysis on the basis of cohort studies, and the conclusion could proved causality relationship. Second, the analysis based on large sample size, and the conclusions of this study was robust than any single study. Third, the exploratory analyses were performed according to region, study design, gender, PD definition, follow-up duration, adjusted level, and study quality, and the differences between subgroups were compared. Finally, the pooled conclusions in our study was robust and not changed by removing any particular study.

The limitations of this study included: (1) the analysis contained both prospective and retrospective cohort studies, and the selection and recall biases were inevitable; (2) the severity of PD were not addressed, which could affect the strength for the associations of PD with the risk of cardiovascular outcomes and all-cause mortality; (3) there were significant heterogeneity across included studies for cardiovascular outcomes and all-cause mortality, which not fully explained by sensitivity and subgroup analyses; (4) the adjusted factors across included studies are differing, which play an important role on the risk of cardiovascular outcomes and all-cause mortality; and (5) inherent limitations for meta-analysis of published data, including inevitable publication bias and restricted detailed analyses.

## Conclusions

This study found PD were associated with an increased risk of MACEs, CHD, MI, stroke, cardiac death, and all-cause mortality. Moreover, region, study design, PD definition, follow-up duration, and study quality might affect the strengths for the associations of PD with the risk of CHD, stroke, and cardiac death. Thus, individuals with PD should be early identified to prevent the progression of cardiovascular outcomes and all-cause mortality. Further large-scale prospective studies should be performed to assess whether the treatments for PD could reduce the risk of cardiovascular outcomes and all-cause mortality.

## Supporting information

S1 File Sensitivity analysis. (DOCX)

S2 File Funnel plot. (DOCX)
