# Supplementary material for: Periodontal disease and subsequent risk of cardiovascular outcome and all-cause mortality: A meta-analysis of prospective studies
Source: PLoS One. 2023 Sep 8;18(9):e0290545. doi: 10.1371/journal.pone.0290545 (PMC10490928; doi:10.1371/journal.pone.0290545)
Supplement: S1 File — (DOCX) [file pone.0290545.s001.docx]

S1 File. Sensitivity analysis


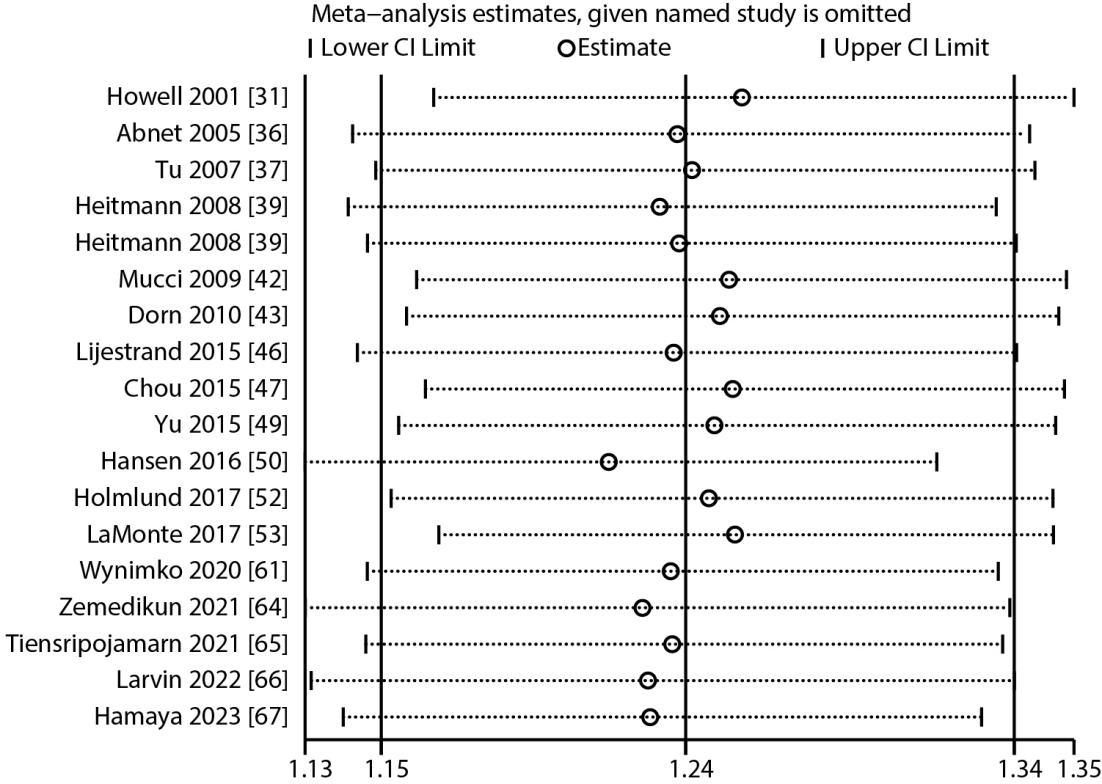


Figure S1. Sensitivity analysis for the association of periodontal disease with the risk of major adverse cardiovascular events.


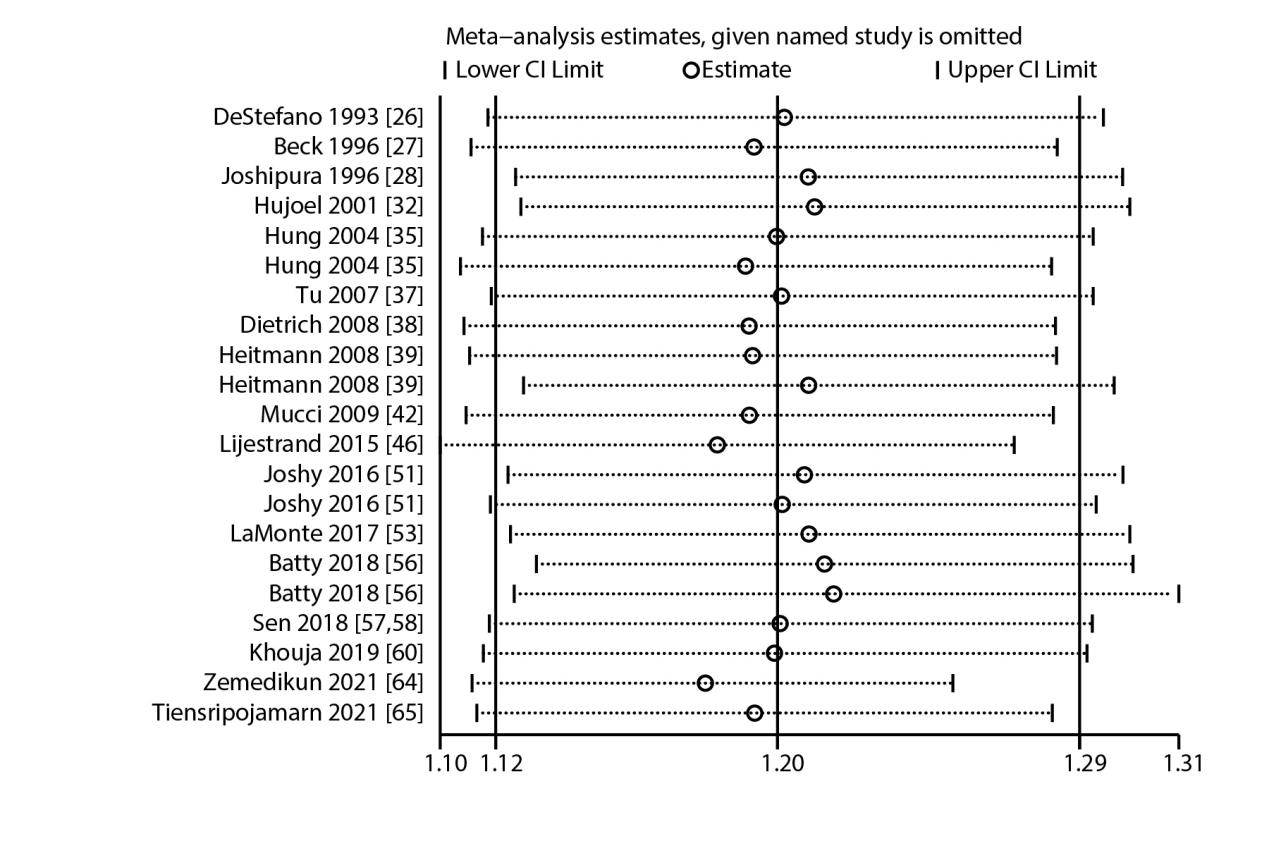


Figure S2. Sensitivity analysis for the association of periodontal disease with the risk of coronary heart disease.


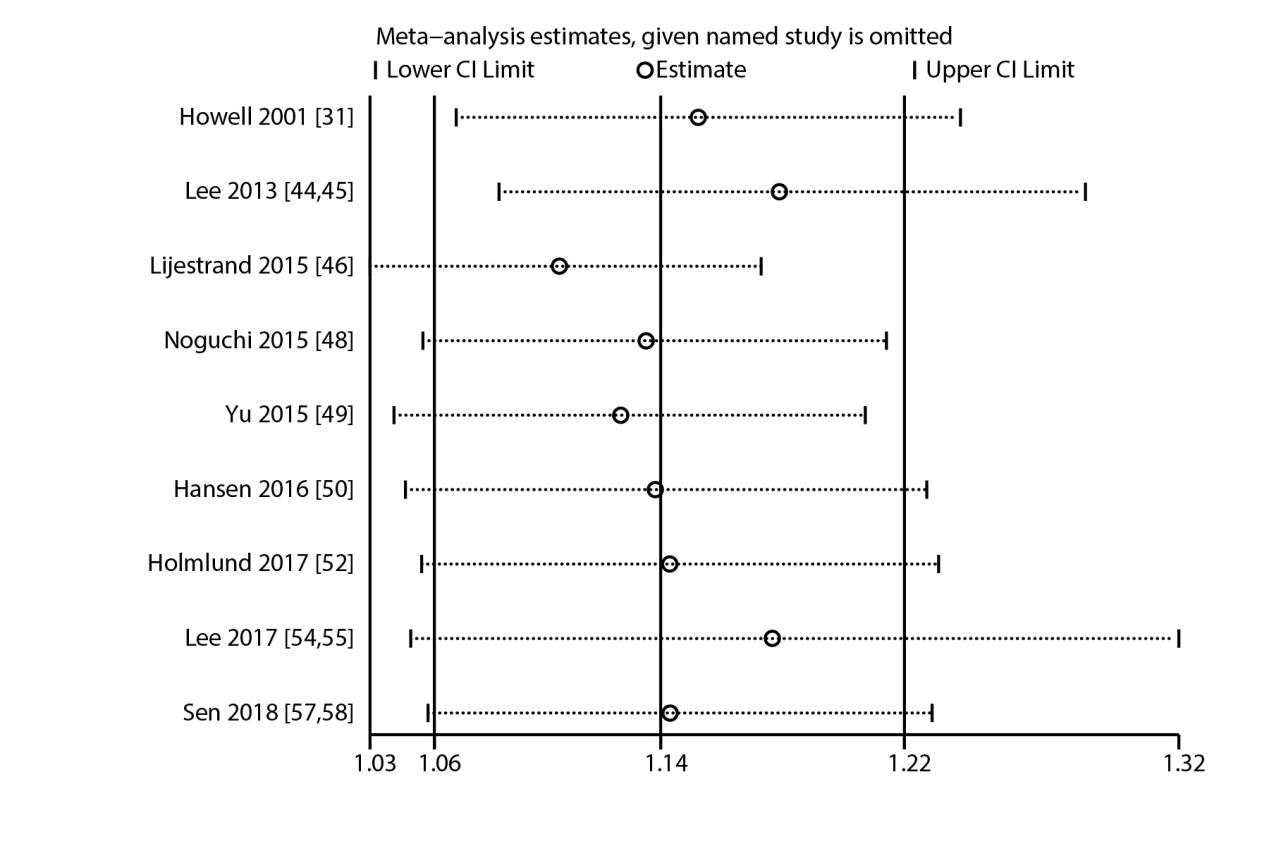


Figure S3. Sensitivity analysis for the association of periodontal disease with the risk of myocardial infraction.


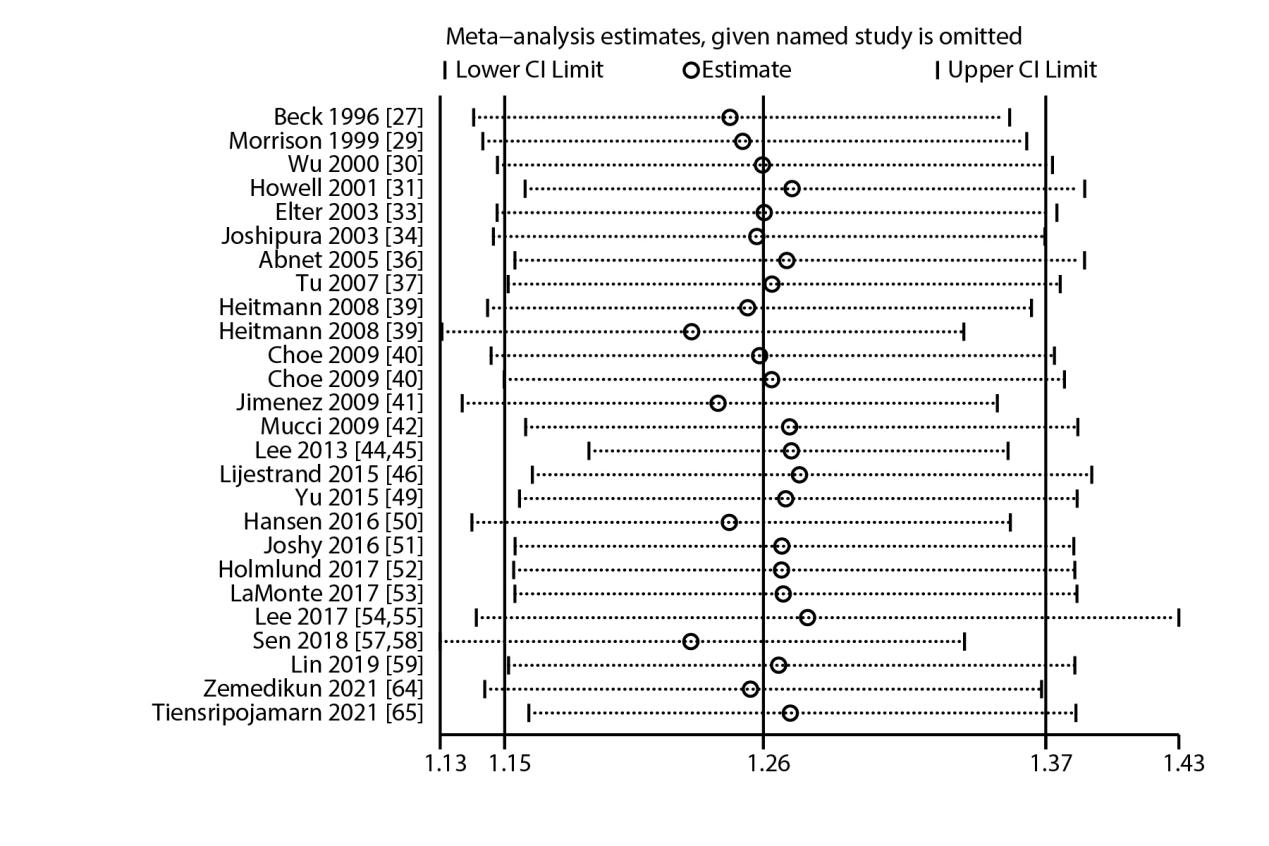


Figure S4. Sensitivity analysis for the association of periodontal disease with the risk of stroke.


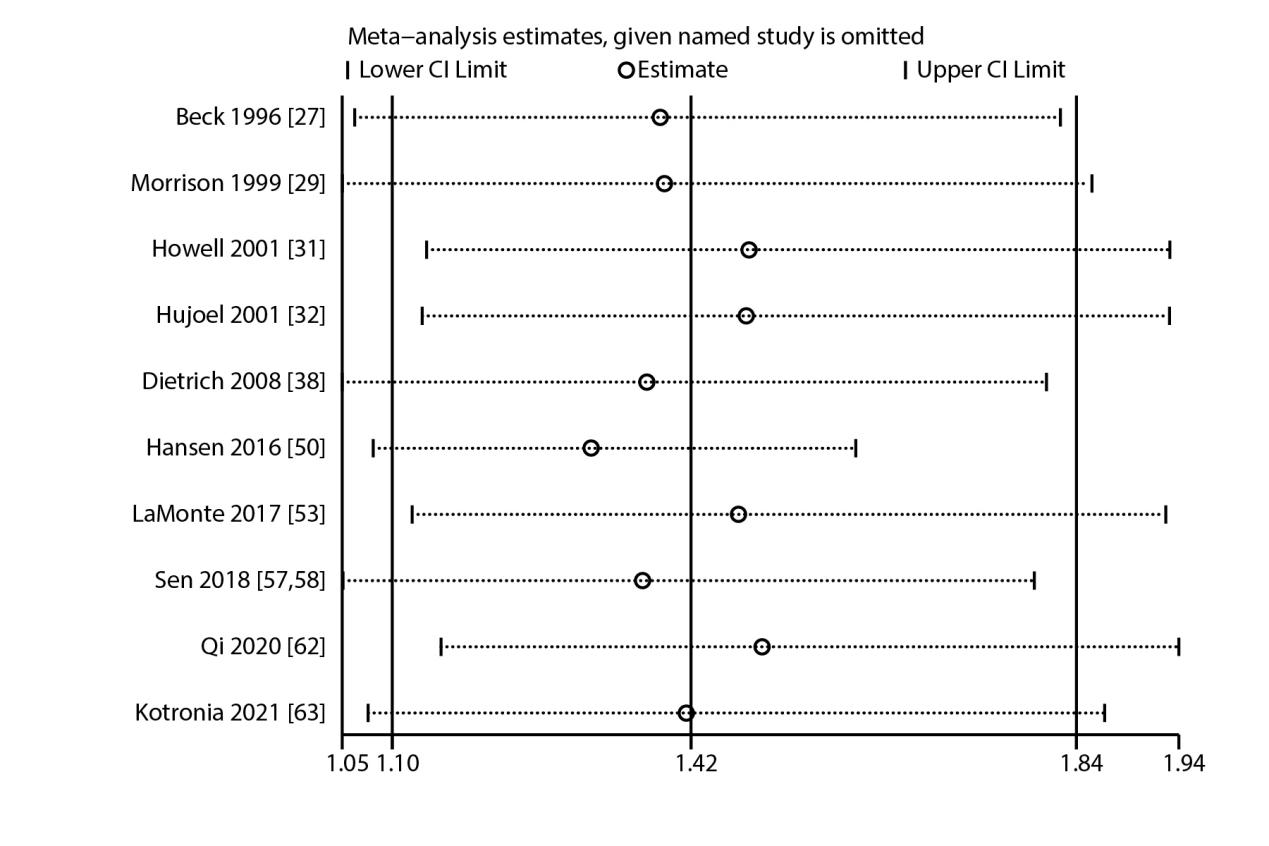


Figure S5. Sensitivity analysis for the association of periodontal disease with the risk of cardiac death.


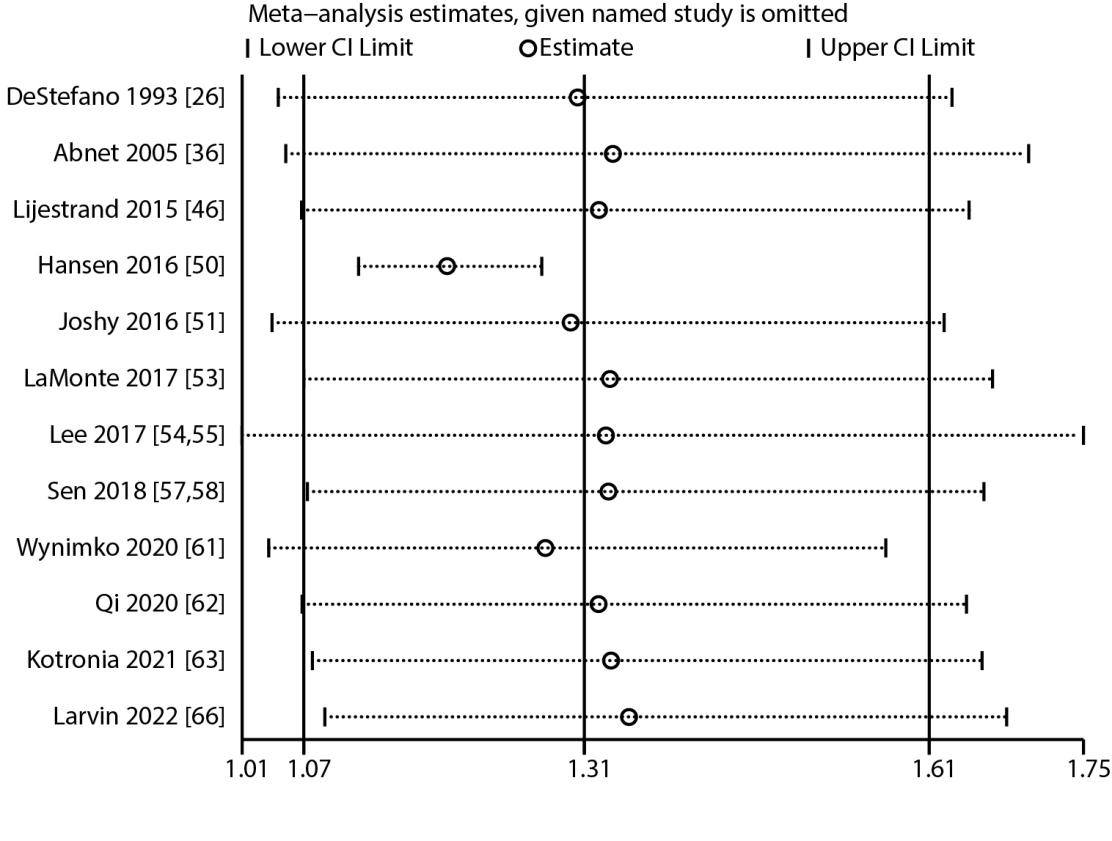


Figure S6. Sensitivity analysis for the association of periodontal disease with the risk of all-cause mortality.
